# From agricultural use of sewage sludge to nutrient extraction: A soil science outlook

**DOI:** 10.1007/s13280-016-0816-3

**Published:** 2016-09-20

**Authors:** Holger Kirchmann, Gunnar Börjesson, Thomas Kätterer, Yariv Cohen

**Affiliations:** Department of Soil and Environment, Swedish University of Agricultural Sciences, P.O. Box 7014, 75007 Uppsala, Sweden

**Keywords:** Ash, Long-term field experiments, Metals, Mono-ammonium phosphate, Soil biology, Urban–rural nutrient recycling

## Abstract

The composition of municipal wastewater and sewage sludge reflects the use and proliferation of elements and contaminants within society. In Sweden, official statistics show that concentrations of toxic metals in municipal sewage sludge have steadily decreased, by up to 90 %, since the 1970s, due to environmental programmes and statutory limits on metals in sludge and soil. Results from long-term field experiments show that reduced metal pollution during repeated sewage sludge application has reversed negative trends in soil biology. Despite this Swedish success story, organic waste recycling from Swedish towns and cities to arable land is still limited to only about 20 % of the total amount produced. Resistance among industries and consumers to products grown on land treated with sewage sludge may not always be scientifically grounded; however, there are rational obstacles to application of sewage sludge to land based on its inherent properties rather than its content of pollutants. We argue that application of urban organic wastes to soil is an efficient form of recycling for small municipalities, but that organic waste treatment from large cities requires other solutions. The large volumes of sewage sludge collected in towns and cities are not equitably distributed back to arable land because of the following: (i) The high water and low nutrient content in sewage sludge make long-distance transportation too expensive; and (ii) the low plant availability of nutrients in sewage sludge results in small yield increases even after many years of repeated sludge addition. Therefore, nutrient extraction from urban wastes instead of direct organic waste recycling is a possible way forward. The trend for increased combustion of urban wastes will make ash a key waste type in future. Combustion not only concentrates the nutrients in the ash but also leads to metal enrichment; hence, direct application of the ash to land is most often not possible. However, inorganic fertiliser (e.g. mono-ammonium phosphate fertiliser, MAP) can be produced from metal-contaminated sewage sludge ash in a process whereby the metals are removed. We argue that the view on organic waste recycling needs to be diversified in order to improve the urban–rural nutrient cycle, since only recycling urban organic wastes directly is not a viable option to close the urban–rural nutrient cycle. Recovery and recycling of nutrients from organic wastes are a possible solution. When organic waste recycling is complemented by nutrient extraction, some nutrient loops within society can be closed, enabling more sustainable agricultural production in future.

## Introduction

One prerequisite for sustainable agriculture is efficient recirculation of plant nutrients. In this paper, we report on the progress made during recent decades in Sweden to improve recirculation of the nutrients present in sewage sludge to arable land. Soil data from long-term field experiments with repeated application of sewage sludge show that environmental efforts to reduce metal pollution of land via sewage sludge have reversed negative trends in soil biology. However, there are other constraints limiting recirculation of nutrients. For large-scale production of sewage sludge in cities, a shift from direct waste recycling to clean nutrient recycling may be required.

## Decreasing metal concentrations in Swedish sewage sludge—A success story

In municipal wastewater treatment plants, human excreta are mixed with other effluents containing metals, organic residues, pharmaceuticals and pathogens (e.g. Östman et al. 2014). Pollutants in sewage sludge can limit its agricultural use. Depending on legislation, rules, attitudes and risk perceptions, use of sewage sludge in agriculture varies between countries. Land application is widely used in France, Spain and the UK, scarcely in Flanders and not practised in the Netherlands and Switzerland where sludge is incinerated. In Greece, Malta and Romania, landfilling is the dominant disposal option.

Since 1986, land application of sewage sludge within the European Union has been governed by Council Directive No. 86/278/EEC. It prescribes prior testing of sludge and soil, application of sewage sludge exceeding critical concentrations of pollutants to agricultural soil is prohibited, and soils exceeding permissible concentrations cannot receive sewage sludge. This Directive has been implemented into the national legislation of member states, most of which have set lower limits than that prescribed in the Directive (Mininni et al. 2015), in order to protect soils and reduce possible emissions (e.g. Pacyna et al. 2006; Thevenon et al. 2011).

Metal concentrations in sewage sludge reflect the amount and type of metals emitted over time by society. Agricultural use of sewage sludge entails transfer of heavy metals and pollutants to arable land, and regular application can elevate metal concentrations in soil to levels toxic to soil microorganisms and affect biological processes (Giller et al. 1998). Application of high concentrations of metals via sewage sludge can reduce the size of the soil microbial biomass (e.g. Brookes and McGrath 1984; Witter et al. 1993) and the activity of nitrogen-fixing bacteria, both free-living forms and those acting in symbiosis with roots (e.g. McGrath et al. 1988; Mårtensson and Witter 1990).

Disruption of soil microbial processes due to heavy metal application via sewage sludge has forced legislators to impose strict regulations on agricultural use of sewage sludge at national (e.g. Sweden: SNFS 1994) and regional levels (e.g. EC 2001, Chap. 5). Reducing emissions of pollutants to wastewater (SWWA 2014) and lowering the permissible concentrations of metals in sludge (NV 2013) have been the guiding principles in work to control and reduce heavy metal pollution of arable soils.

### Decline in heavy metals in Swedish sewage sludge since 1970

**Fig. 1:**
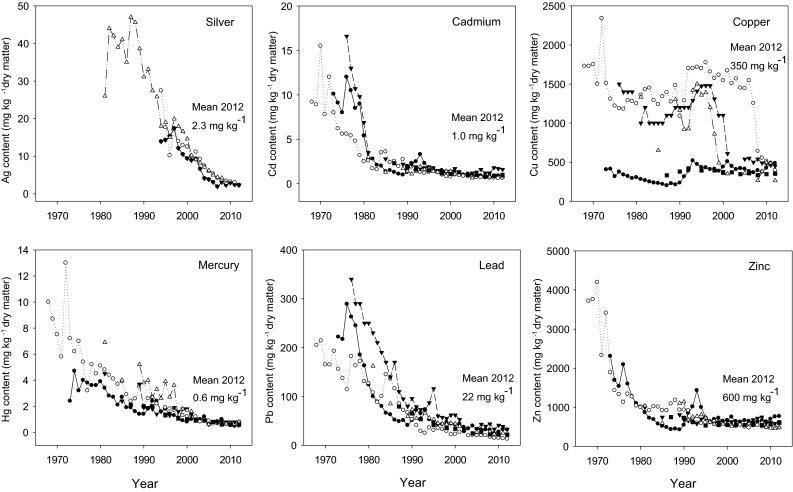
Decline in the concentrations of silver, cadmium, copper, mercury, lead and zinc in Swedish sewage sludge over time (1970–2010). Mean data for silver are not available (*open circle* sludge from Uppsala; *filled circle* sludge from Gothenburg; *open triangle* sludge from Stockholm; *inverted filled triangle* sludge from Malmö/Lund; *filled square* mean data on Swedish sewage sludge). Data taken from Statistics Sweden (SCB 1987, 1990, 1992, 1995, 2012) with additional information from Sveder (2002), Ernst-Olof Swedling, Kungsängsverket, Uppsala (pers. comm. 1 Dec. 2011), Lars Nordén, Gryyab, Gothenburg (pers. comm. 14 April 2014) and Mats Thuresson, Stockholm County Administrative Board (pers. comm. 1 March 2013)

Graphs showing changes in the concentrations of heavy metals in sludge from sewage treatment plants in the four largest cities in Sweden (Stockholm, Gothenburg, Malmö and Uppsala) and mean values for a large number of plants recorded in official statistics in the period 1970–2010 illustrate trends over time (Fig. 1). Trends for four non-biogenic metals (silver (Ag), cadmium (Cd), mercury (Hg) and lead (Pb)) and two essential elements (copper (Cu) and zinc (Zn)) are demonstrated to represent changes in metal concentrations in sewage sludge.

Inputs of metals to urban wastewater occur from three sources: domestic, commercial and runoff (EC 2001, Chap. 1). Except for Cu from plumbing and Zn from roofing, runoff is not a major contributor of toxic elements to sewage systems, whereas domestic wastewater contributes significantly to the metal load, mainly through input from faeces. Faecal matter contains, on average, 2 mg Cd, 70 mg Cu, 10 mg Pb and 250 mg Zn per kg dry matter (EC 2001, Chap. 1). Other main sources of metals in domestic wastewater are bodycare products, pharmaceuticals, cleaning products and liquid wastes. However, complete identification of all sources for some metals is still lacking. It is evident that the concentrations of major heavy metals in sewage sludge have declined considerably over time (Fig. 1). The major reasons for this are discussed briefly below.

*Silver* is used in jewellery, cutlery, coins, electrical contacts and conductors. Moreover, until a couple of years ago, silver chloride suspensions were commonly used for photography, causing significant waste flows. Replacement of X-rays and black and white photographs with digital images can explain the rapid decrease in Ag concentrations from around 40 to on an average 2.3 mg Ag per kg dry sludge since the 1990s (Diener and Palme 2012), see Fig. 1. However, this decrease has halted during recent years, probably due to emerging textiles containing silver as an antibacterial compound that is leached out with laundry water (KEMI 2011a).

*Cadmium* was previously widely used for coating metal surfaces to prevent corrosion, in rechargeable nickel–cadmium batteries, in electrical components, etc. Due to adverse effects on the environment and human health, the use of Cd was banned in 1985 in Sweden (SFS 1985) and is restricted in Europe under the REACH regulations (REACH 2006). The decline in concentration in sewage sludge between 1970 and 1990 from about 10 to 2 mg Cd per kg dry matter was probably due to the result of this ban on cadmium. A further decline to a mean value of 1 mg Cd per kg dry matter can mainly be attributed to treatment of exhaust fumes from combustion plants (Pacyna et al. 2009), reducing the deposition load.

*Copper*, which is an essential element for organisms, is commonly used as a pure metal in electrical wires, roofing, plumbing and industrial machinery (Lander and Lindeström 1998). In certain areas, drinking water releases Cu from plumbing, causing high Cu contents in wastewater and sewage sludge. The steep declines in Cu concentrations in sewage sludge within a few years (Fig. 1) are explained by changes in the composition of drinking water to limit Cu dissolution. Mean values of 350 mg Cu per kg sludge dry matter can be regarded as representative for Sweden.

*Mercury* and most of its chemical forms can cause serious health and environmental damage. In Sweden, a ban on the use of Hg in technical devices and electrical components was introduced in the 1990s, exports were prohibited in 1997 and a total ban on the use of Hg was implemented in 2009 (KEMI 2011b). Collection of Hg (e.g. thermometers, amalgam separators at dental practices) for appropriate disposal, measures to limit emissions from waste, coal and other combustion plants to the atmosphere and finally a complete ban on use have had a positive effect. Consequently, mean values of around 4 mg in the 1970s have dropped to 0.6 mg Hg per kg sludge dry matter in 2012.

*Lead* was formerly used as an additive to petrol, before strict exhaust emission regulations were implemented in Sweden (Regeringens Proposition 1985/86). Since the ban on Pb as a petrol additive, concentrations have fallen from around 250 mg to on average 22 mg Pb per kg sludge dry matter. Replacement of old lead pipes for drinking water with modern pipes may be another reason for the lower concentrations in sludge.

*Zinc*, an essential element of enzyme systems in organisms, is mainly used in industry to plate iron or steel (galvanisation) to prevent corrosion. Zinc enters into wastewater from a number of sources, e.g. galvanised iron plumbing, deodorants and antidandruff shampoos containing zinc chloride, antifouling paints with zinc pyrithione and runoff from galvanised roofs. The exponential decrease in Zn in sewage sludge since 1970, from around 3000 to 600 mg Zn per kg sludge dry matter, may have a number of explanations, but the decline in atmospheric Zn emissions from combustion plants and industries, and the reduction in industrial outflows are probably the main reasons.

In summary, the quality of sewage in terms of heavy metal content has greatly improved over the past 20 years. Concentrations in sewage sludge of the six metals discussed above have decreased by on an average 85 %, showing that environmental policies followed by measures to control metal emissions in Swedish society have been very effective. According to the Swedish legislation on metal input to arable land (SNFS 1994), the annual maximum metal supply per hectare is 0.75 g cadmium, 300 g copper, 1.5 g mercury, 25 g lead and 600 g zinc. Based on actual mean metal concentrations in Swedish sewage sludge (Fig. 1), cadmium in sewage sludge (1.0 mg Cd per kg dry matter) is identified limiting application of sewage sludge to about 750 kg dry matter per hectare and year.

### Effects of heavy metals on soil biology in long-term field experiments with sewage sludge

In four field trials in Sweden (see Börjesson et al. (2012) for experimental description), sewage sludge has been applied repeatedly over a long period. The Ultuna trial at Uppsala has been running since 1956, the Igelösa and Petersborg trials in southern Sweden since 1981 and the Lanna trial in south-western Sweden since 1996. The sludge is obtained from nearby wastewater plants and applied at rates amounting to 1–4 Mg sludge dry matter per hectare and year (Table 1). These amounts are much higher than the Swedish limit of 700 kg dry mass, corresponding to about 0.75 g of cadmium and 22 kg phosphorus (P) per hectare and year.

**Table 1:** Yield, *N* use efficiency, soil balances of *N* and *P* and bulk density in the four Swedish long-term field experiments with sewage sludge

Site, years and soil treatments	Mean yield	Nutrient application		Nutrient removal		*N* use efficiency^a^	Δ*N* in soil	Δ*P* in soil	Bulk density (2009)
		*N*	*P*	*N*	*P*				
	kg ha^−1^	kg ha^−1^ year^−1^		kg ha^−1^ year^−1^		% of added *N*	kg ha^−1^ year^−1^	kg ha^−1^ year^−1^	kg dm^−3^
Ultuna 2002–2009									
Control	3329	0	20	35	8	–	−35	+12	1.43
Mineral fertilised	7176	80	20	95	15	75	−15	+5	1.28
Sewage sludge treated, 4 Mg C ha^−1^ every 2nd year	9719	276	233	146	19	40	+131	+215	1.02
Lanna 1996–2009									
Control	1316	0	0	19	4	–	−19	−4	1.38
Mineral fertilised	3407	80	20	57	11	55	+23	+9	1.36
Sewage sludge treated, 8 Mg dry matter ha^−1^ every 2nd year	3450	236	194	63	12	19	+173	+183	1.30
Igelösa 2006–2009									
Control	4038	0	0	96	26	–	−96	−26	n.a.
Mineral fertilised	8010	128	19	154	38	45	−26	−19	n.a.
Sewage sludge treated, 12 Mg dry matter ha^−1^ every 4th year	4838	105	171	110	29	13	−5	+141	n.a.
Sewage sludge + *N* treated, 12 Mg dry matter ha^−1^ very 4th year	7698	232	190	161	38	28	+71	+152	n.a.
Petersborg 2006–2009									
Control	3308	0	0	79	21	–	−79	−21	1.68
Mineral fertilised	6850	139	23	167	41	63	−28	−18	1.59
Sewage sludge treated, 12 Mg dry matter ha^−1^ every 4th year	4235	132	105	98	25	14	+34	+80	1.61
Sewage sludge + *N* treated, 12 Mg dry matter ha^−1^ every 4th year	7330	271	128	196	43	43	+75	+84	1.56

*n.a.* not analysed

^a^Nitrogen use efficiency was based on the difference calculation: (*N* removal_treatment_ − *N* removal_control_)/*N* input × 100

Since the concentrations of metals in sludge began to decrease during the 1970–1980s (see Fig. 1), only the Ultuna trial (start 1956) has received strongly metal-polluted sludge. In the other trials, starting 25–41 years later, most metal concentrations in sewage sludge had already declined. Consequently, metal concentrations in soils at these sites are generally much lower than in soil samples from the Ultuna trial (Table 2). For example, the Cd concentration in Uppsala sewage sludge was 9.1 mg per kg in 1972 and decreased to 0.65 mg per kg in 2009. However, in the same period, the concentrations of Cu and Zn increased significantly in sludge-amended plots at all sites compared with plots receiving only mineral fertiliser (Table 2). Mean yearly metal increase in soil over all sites was 1.9 % for Cd, 9 % for Cu, 1.2 % for Pb and 2.2 % for Zn. As the Cu and Zn concentrations in sewage sludge are much higher than those of Cd, Pb and Hg (Fig. 1), Cu and Zn concentrations will continue to increase in soil over time even when ‘low-metal’ sludge is applied.

**Table 2:** Mean concentrations of cadmium, copper, lead and zinc in soils (mg kg^−1^ soil dry weight) due to long-term sewage sludge application, compared with the use of mineral fertiliser, in four Swedish field experiments. (Data from Börjesson et al. 2012)

Site, start and sampling year	Cadmium		Copper		Lead		Zinc	
	Sewage sludge	Fertiliser	Sewage sludge	Fertiliser	Sewage sludge	Fertiliser	Sewage sludge	Fertiliser
Ultuna, 1956–2010	0.73	0.24	196.0	27.8	41.0	21.6	271.0	87.6
Lanna, 1996–2010	0.14	0.12	20.8	8.9	14.4	14.1	83.1	65.6
Igelösa, 1981–2010	0.34	0.30	25.8	15.3	17.3	16.3	58.0	47.5
Petersborg, 1981–2010	0.26	0.24	21	9.4	14.0	13.5	45.3	38.0

**Fig. 2:**
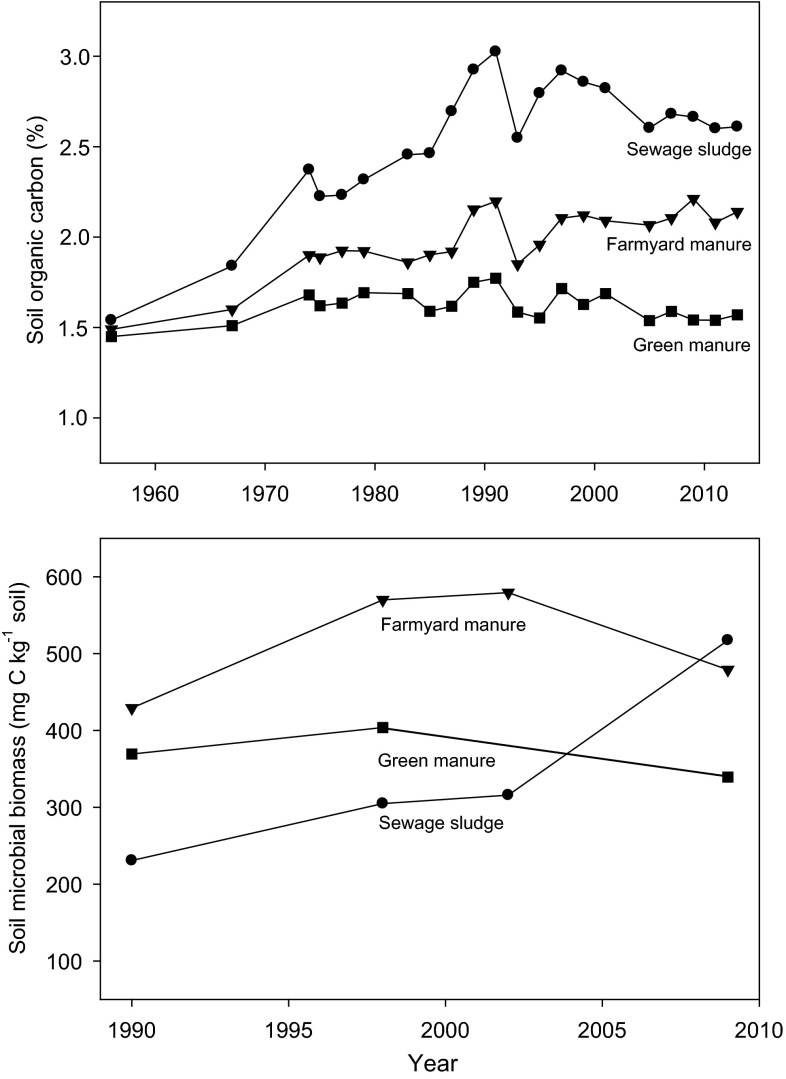
Changes in (*upper diagram*) soil organic carbon and (*lower diagram*) soil microbial biomass in the topsoil (0–20 cm) of the Ultuna field experiment over time. Note the reverse in trend in the sewage sludge-treated soil since 1990 to a decline in organic carbon and an increase in microbial biomass. Estimates of soil microbial biomass carbon were derived from previous publications using different methods. The following conversion factors were applied to obtain the same units of soil microbial biomass: 1990: ATP (Witter et al. 1993), 1 μg *C* = 170.94 μg ATP (Tate and Jenkinson 1982); 1998: dsDNA (Marstorp et al. 2000), 1 μg *C* = 6.0 μg DNA (Joergensen and Emmerling 2006); 2002: SIR (Enwall et al. 2007), 1 μg *C* = 40.04 ml CO_2_ h^−1^ + 0.37 (Anderson and Domsch 1978); we assumed that 1 mg CO_2_–C is equivalent to 1.868 ml CO_2_ under standard conditions (0 °C and 1 atm). 2009: PLFAs (Börjesson et al. 2012), 1 μg C = 5.8 nmol PLFA (Joergensen and Emmerling 2006)

In the Ultuna trial, addition of sewage sludge and farmyard manure has caused an increase in soil carbon concentrations over time, while carbon concentrations have remained relatively constant in the treatment where green manure is added (Fig. 2). It is interesting to note that the upward trend in soil organic carbon in the sludge treatment ceased in the period 1990–1997 and thereafter levelled off, and has even declined since the end of the 1990s (Fig. 2). This decline suggests that degradation of soil organic matter has increased since 1997. As the same amount of sludge C has been added at each application time, the organic matter composition of sewage has remained unchanged, and no major technical changes have been made in the treatment plant; the declining trend in soil organic matter can only be due to increasing decomposition.

Furthermore, the decline in soil organic carbon has been accompanied by an increase in soil microbial biomass (Fig. 2). In the Ultuna trial, the microbial biomass has been investigated with four different methods in independent studies over the years. In order to compare data obtained using different methods, conversion factors given in the literature (see Fig. 2) were used here to estimate the microbial biomass. Data from studies involving ATP measurements (Witter et al. 1993), dsDNA measurements (Marstorp et al. 2000), substrate-induced respiration (SIR) (Enwall et al. 2007) and phospholipid fatty acids (PLFA) (Börjesson et al. 2012) were recalculated to a common unit, biomass per dry mass of soil. Since the methods have changed over time, estimates of absolute changes in biomass due to individual treatments are associated with high uncertainty, but the comparison of biomass estimates between treatments in relative terms should be robust. Moreover, the biomass in the treatment receiving green manure, in which soil organic carbon concentrations are close to steady state, has remained relatively constant over time. This indicates that biomass estimates are also comparable between methods in absolute terms. Since the biomass studies were performed at regular intervals over the years (1990, 1998, 2002 and 2009), it is possible to identify trends (Fig. 2). The amount of microbial biomass in the sewage sludge treatment increased between 1990 and 1998, and between 2002 and 2009. The amount was twice as large in 2009 as in 1990. The late 1990s onwards was also the period when soil organic carbon declined in the sludge-treated soil and when sludge with much lower metal concentrations was applied (Fig. 1).

Apart from the metals discussed above, sewage sludge contains a large number of other metals (Eriksson 2001) not considered in this paper. However, comparisons show that metal concentrations in Swedish sewage sludge are lower than those in sludge of other European countries (IWA Waterwiki 2016). In addition to metals, sewage sludge usually also contains a range of organic pollutants; however, most of these do not directly impact on soil microbes or can remain unavailable to microbes in soil pores for decades (Bergström and Stenström 1998).

In summary, the results indicate that the microbial biomass in soils was stressed until about 1990 by metal addition with sewage sludge. Thereafter, the microbial population recovered to its normal size due to lower metal loads, and the proportion of soil organic carbon reached a similar magnitude as in non-metal-polluted soil (Fig. 2).

## Constraints that limit recycling of sewage sludge and agricultural use

Despite impressive improvements in sewage sludge quality, producing safe and clean wastes is not sufficient to achieve recycling. There are two inherent characteristics that restrict recycling of urban organic wastes: (i) low plant availability of the nutrients applied and (ii) high water and low nutrient content and consequently large waste volumes per nutrient to be distributed.

### Sewage sludge has a low fertiliser value, and its contribution to soil organic matter is small

Nitrogen and phosphorus are the most valuable nutrients in sewage sludge. Other water-soluble nutrients such as potassium (K), calcium (Ca) and magnesium (Mg) are less efficiently removed during wastewater treatment, and concentrations in sewage sludge are lower than in other organic manures (Sjöquist and Wikander-Johansson 1985; Kirchmann and Witter 1992).

About 10 % of the total nitrogen in sewage sludge is present as ammonium nitrogen (Sjöquist and Wikander-Johansson 1985), which is plant available, while the majority of the nitrogen (~90 %) is present in organically bound forms that need to be mineralised to become plant available. Similarly, about 10 % of total phosphorus in sewage sludge is present in water-soluble and easily extractable forms, and the rest is strongly bound to iron (Fe) or aluminium (Al), as shown by sequential fraction of sewage sludge (Xu et al. 2012). This means that the crop availability of these nutrients can be expected to be lower than that of mineral fertilisers, as also shown in pot and field trials (e.g. Delin 2015; Krogstad et al. 2015). Similarly, crop utilisation of micronutrients from sewage sludge has been found to be very low (Hamnér and Kirchmann 2015).

When similar amounts of nitrogen were applied with sewage sludge as with mineral fertiliser, crop yields increased by 19–28 % compared with the unfertilised control, but yields doubled in the plots with mineral nitrogen (Table 1). This confirms the limited plant availability of nitrogen in sewage sludge, as shown by chemical analysis (Sjöquist and Wikander-Johansson 1985). Nitrogen use efficiency for sewage sludge in the study period varied between 13 and 40 % (Table 1). The use efficiency for phosphorus in sewage sludge was not calculated, as the amounts of P added with sludge were too high in relation to crop demand, which would result in misleading interpretations.

Applying large amounts of sewage sludge to arable land over a long time, which is not permitted according to Swedish law, improved soil structure. Soil bulk density values decreased in sludge-treated soil, indicating higher soil porosity and better root proliferation (Table 1). In fact, decreasing soil bulk density values in sludge-treated soils were positively correlated with nitrogen use efficiency from sewage sludge (*r* = 0.93).

Despite positive soil structural effects, the amounts of sewage sludge that can be added to arable land are rather limited, both when all sludge is distributed equitably on agricultural land and when the permissible quantity per hectare is applied. About 214 000 Mg dry matter of sewage sludge is produced in Sweden every year (SCB 2012). Based on the total area of arable land in Sweden (2.5 million ha), the total quantity would correspond to an application rate of 86 kg dry matter, equivalent to 24 kg organic carbon, per hectare and year. The permitted quantity is about 700 kg dry matter, corresponding to 196 kg organic carbon. Assuming that 41 % of the carbon added with sludge is stabilised in soil (Kätterer et al. 2011), the net effect of sludge addition on soil organic carbon would be at least 10 kg and at most 80 kg per hectare and year. For comparison, organic carbon added through crop residues (above and belowground) amounts to 1000–3000 kg per hectare and year, with a net supply after decomposition of 200–600 kg soil organic matter. Thus, normal rates of sewage sludge application add a small amount of organic matter to arable soil, with minor or no effects on soil structure.

### Organic waste accumulation in cities is the main bottleneck for recycling to arable land

The 20th Century witnessed rapid urbanisation in the world. The proportion of urban population increased from 13 % in 1900 to 29 % in 1950 to reach 50 % in 2009 (United Nations 2010). Furthermore, future population growth is expected to occur mainly in urban areas, with the urban population projected to increase from 3.4 billion in 2009 to 6.3 billion in 2050 (GeoHive 2010). Urbanisation means that food is accumulated in cities, which therefore become hot spots for nutrients and other elements (Færge et al. 2001; Warren-Rhodes and Koenig 2001; Grimm et al. 2008). However, food is mainly produced in remote agricultural areas supplying urban areas. In order to close the urban–rural nutrient cycle, nutrients transported to urban areas in the form of food would need to be returned to remote arable land where the food was produced. This imposes two specific challenges:

1. To avoid nutrient enrichment of arable land surrounding cities and nutrient depletion of remote arable land, long-distance transportation and equitable redistribution are required.
2. Recycling should not lead to contamination of arable soils.

One major factor constraining organic waste recycling to arable land is the high water and low nutrient content. For example, dewatered sewage sludge contains 70–90 % water, compost 55–65 % and biogas residues 90–95 %. Therefore, large volumes of organic waste need to be stored, handled and transported before being recycled. For comparison, organic wastes have higher water and lower nutrient content than staple food crops (Table 3), whereas inorganic wastes, such as ash, contain more nutrients than organic wastes. Long-distance transportation of urban organic wastes is costly, and beyond a certain transport distance, the costs will exceed the revenue obtainable from nutrients in the form of mineral fertilisers. This can explain why only minor proportions of organic wastes from major cities in the world are recycled, and only to peri-urban areas (e.g. Færge et al. 2001; Donatello et al. 2010).

### Conditions for sustainable nutrient recycling

Closing nutrient cycles, i.e. re-circulating nutrients removed through harvested crops back to agricultural land, is an important target for sustainable agriculture. One major precondition for nutrient recycling is to produce as clean and safe municipal wastes as possible. The absence of environmental pollutants in wastes is one critical feature, but there are additional conditions that must be fulfilled to achieve fully functioning recycling (Table 4). These include the following: (1) cost-effective waste treatment and transport, allowing equitable redistribution of nutrients to arable land; (2) significant fertiliser value of products that can replace mineral fertilisers; and (3) ‘safe and clean’ wastes that have no adverse effect on crops, soils or the environment. Thus, managing organic wastes according to the environmental target ‘safe and clean’ only is not the way forward, as all the three conditions must be fulfilled to close the nutrient cycle.

**Table 3:** Water and phosphorus (P) content in mineral fertiliser, crops and urban wastes. (Data from Svanberg 1971; Koivistonen 1980; Sjöquist and Wikander-Johansson 1985; Kirchmann and Pettersson 1995; Eklind et al. 1997; Cohen 2009)

Type of product	Water content (% of wet weight)	Phosphorus content (kg P Mg^−1^ wet weight)
Mineral P fertilizer		
Ammonium phosphate	<1	220
Struvite (magnesium ammonium phosphate hexahydrate)	44	8
Crops		
Oil-seeds	16	6
Peas, beans	16	3.6
Cereal grains	16	3.5
Forage	25	3.2
Potatoes	76	0.5
Sugar beet	79	0.3
Urban wastes		
Ash from sewage sludge	1–3	90
Sewage sludge	70–90	7
Compost	55–65	1.5
Biogas residues	90–95	0.7
Human urine	99	0.2
Waste water	99	0.01

**Table 4:** Conditions and product characteristics required to achieve sustainable nutrient cycling in society

Cost-effective recycling
Energy- and resource-efficient waste treatment Easily spreadable products allowing equitable redistribution—low water and high nutrient content
High fertiliser value of products that can replace mineral fertiliser
High plant availability of nutrients over the short term Low emissions and leaching losses
Safe and clean products that have no adverse effects on crops, soils or the environment
Low levels of organic contaminants Low levels of unwanted metals Low levels of pharmaceuticals Low levels of pathogens

As mentioned above, the high water and low nutrient content in urban organic wastes is limiting for long-distance transportation of large volumes, and the low fertiliser value is an obstacle to recycling. Furthermore, combustion of organic wastes reduces the volume and water content, but metal enrichment in ash restricts the possibility to use ash on arable land. Metal concentrations in sewage sludge ash are 8–9 times higher than in sewage sludge dry matter only allowing an application rate of less than 100 kg per hectare and year. The principal question arises of how to achieve recycling of nutrients from cities back to arable land. We believe that the answer lies in the development of technologies for extracting nutrients from wastes, instead of recycling wastes directly to soil.

## Future waste management in cities—From direct waste recycling to nutrient extraction from ash

### Characterisation of sewage sludge ash

The main treatment applied to sewage sludge in most cities is combustion, with the final product being ash. As sewage sludge is based on renewable biomass, it is a carbon-neutral fuel. There is a trend to pyrolyse sludge and other organic wastes to fuel gases and biochar (Fytili and Zabaniotou 2008). Although biochar from organic residues principally can be used as a soil improving product (e.g. Carlsson et al. 2012; Parvage et al. 2013), metal contents in biochar from sewage sludge are too high (e.g. Song et al. 2014) and similar to ash limiting its use as a soil amendment. Thus, biochar from sewage sludge still needs to be combusted, resulting in ash again. Ash will most probably become the main product of urban organic wastes in cities in future. Through combustion, the total weight of sewage sludge is reduced by approximately 75 % (Kirchmann et al. 1994). Organic compounds are oxidised and released as gases, whereas most metals are enriched in the ash due to low or no volatilisation. For example, zinc in ash of sewage sludge can amount to 5000 mg per kg (Hong et al. 2005), compared with 600 mg per kg in dry sewage sludge. Thus, direct use of ash on arable land is rarely possible without reducing the metal content. Volatilising metals from ash as metal chlorides at high temperature can lower the residual metal concentration, but some metals (e.g. Pb, Zn) still remain above the threshold values for agricultural use (Nowak et al. 2010).

Nitrogen is lost as nitrogen gas during combustion, but nitrogen is not a limited resource on earth and can be recovered from the atmosphere (which contains ~78 % nitrogen) by chemical nitrogen fixation. In fact, the amounts of nitrogen lost during combustion of sewage sludge have little relevance for agriculture. For example, the total amount of sewage sludge produced in Sweden every year contains around 5000 tons of nitrogen, which is equivalent to approximately 2 kg nitrogen per hectare arable land. The nitrogen demand of crops usually varies between 80 and 200 kg per hectare and year.

Concerning potassium, concentrations in sewage sludge are very low (0.1 % K of dry matter), since potassium is generally water soluble and not incorporated into the solid phase (Binnie 1995) and remains below 1 % in sewage sludge ash (Cohen 2009). Recovery of potassium from sludge ash is therefore less relevant.

The main plant nutrient present in sewage sludge is phosphorus. The concentrations of phosphorus in sewage sludge ash (7–13 % P) are similar to those in apatite (12–16 % P), making P recovery highly relevant (Cohen 2009).

### New extraction methods for phosphorus from ash

During the past decade, several technologies for phosphorus recycling from sewage sludge ash, including both pyro- and wet-based processes, have been developed (e.g. Nowak et al. 2010; Easymining 2014). In pyro-based technologies, metals in the ash are partly removed (Nowak et al. 2010) but phosphorus remains in the form of insoluble apatite, which is not plant available and has a low fertiliser value. In wet-based processes, sewage sludge ash is treated with an acid or base to dissolve the phosphorus, which can then be recovered through precipitation as ammonium, calcium, sodium, iron or aluminium phosphate, which means also in forms identical to those in mineral P-fertiliser. For example, the CleanMAP^®^ technology (EasyMining 2014) enables production of fully water-soluble ammonium or calcium phosphate of the highest quality without metal contamination, irrespective of ash quality. Only when phosphorus is extracted without contamination and in concentrated form can safe and equitable redistribution on remote cropping areas cities be achieved.

Of course, treatment of ash for nutrient extraction must be compared against treatment of new resources such as rock phosphate with respect to consumption of chemicals and emissions (Life Cycle Assessment). At present, no details are available for the CleanMAP technology, and only typical features can be outlined. Concerning acid consumption, the quantities needed for dissolution of ash are similar to those for dissolution of rock phosphate. Further, chemicals required for extraction in the CleanMAP technology, for example ammonia, chlorides and sulphides, end up in final product in the form of ammonium phosphate, iron and aluminium chloride and metal sulphides. However, one of the most energy-demanding steps during current phosphorus fertiliser production is water evaporation to convert nutrient solutions from liquid to solid form. In the CleanMAP technology, all recovered compounds are precipitated without water evaporation. Thus, resource consumption and associated emissions through CleanMAP are at least not higher than through other fertiliser production technologies.

## Concluding discussion

Sewage treatment plants are important for human health and the environment. Reducing contamination of sewage sludge with metals and organics is worth the effort, since low pollution of sewage sludge is an indicator of efficient environmental policies to control the use of man-made compounds in society. The efforts undertaken in Sweden to decrease metal loads through application of sewage sludge to soil have resulted in lower stress for soil organisms and increased turnover of soil organic matter. This illustrates that concerted political actions can stop deterioration of soil quality and can lead to environmental success. Thus, in the case of metals, the risk of using sewage sludge in agriculture can be regarded as extremely low, provided that the legislation is followed. Concerning organic chemicals, pharmaceuticals and pathogens present in sewage sludge, the number of such substances is much larger than that of metals (Schoof and Houkal 2005), but the risks related to these pollutants are reported to be low (e.g. Norwegian Scientific Committee for Food Safety 2009). However, occasional outbreaks of the disease salmonellosis caused by sewage sludge (e.g. Sahlström et al. 2006) still illustrate that there can be a risk.

Even if possible risks related to contaminants in sewage sludge can essentially be eliminated in future, specific obstacles to closing the urban–rural nutrient loop when recycling urban organic wastes still remain (see Table 3). Since sufficient arable land is often not available in the vicinity of cities, long-distance transportation of sewage sludge is required for recycling, which is expensive due to the high water and low nutrient content of the sludge. The approach of direct organic waste recycling must therefore be diversified. Direct recycling of urban organic wastes is only valid up to a certain population density, since the larger the city, the more organic waste will have to be combusted. Urbanisation will force a change, with ash being the main urban waste product, and the direct route of organic waste recycling will become increasingly fragmented.

**Fig. 3:**
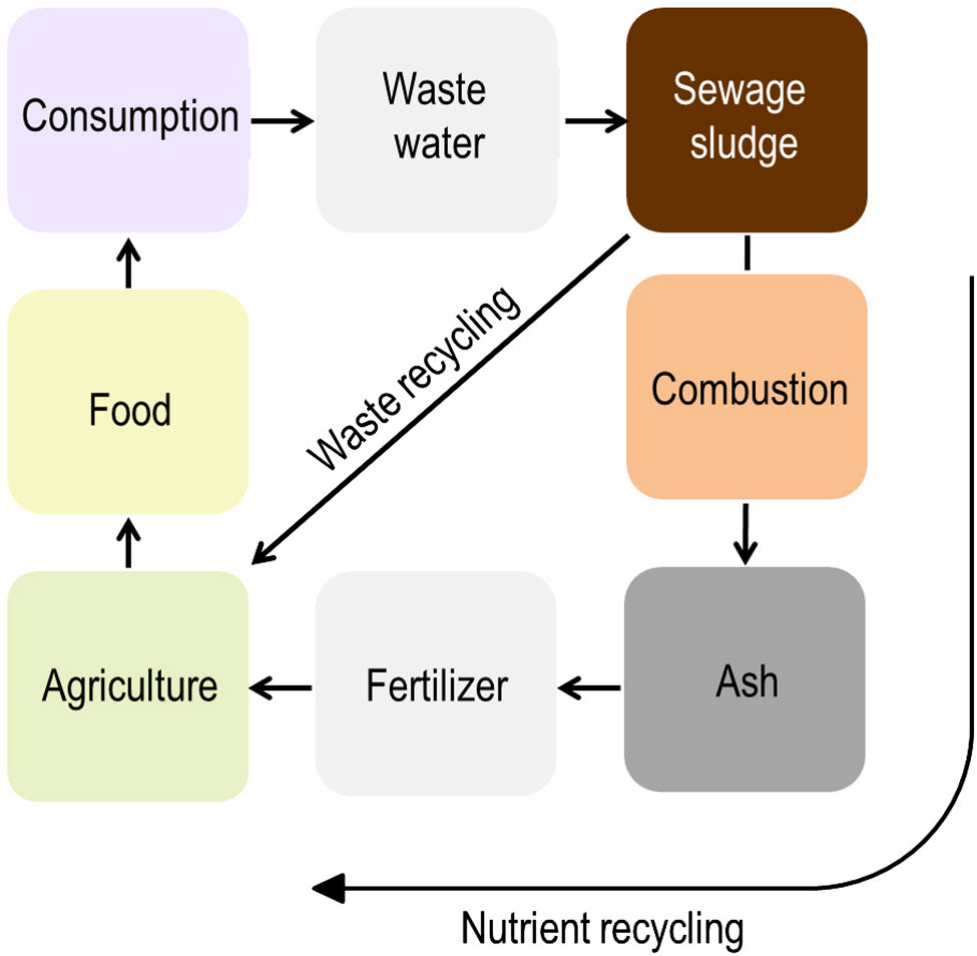
Flow diagram illustrating the additional concept to close nutrient cycling in society: Recycling nutrients from organic waste by additional treatments: combustion, ash extraction and fertiliser production

In order to avoid ash deposition, the development of new technologies treating ash to produce concentrated and clean fertiliser products that can substitute for mineral fertiliser is a possible way forward (Fig. 3). The advantages are apparent: (1) Use of the raw material apatite for phosphorus fertiliser production can be reduced and replaced with ash; (2) extracted fertiliser products can have as high water solubility as those on the market, which means highest nutrient availability for crops; (3) concentrations of unwanted contaminants are very low and; (4) transportation of recycled nutrients back to remote cropland becomes possible, enabling equitable distribution.

In parallel with the development of new nutrient recycling technologies from waste, another challenge is to improve the quality of water effluent from sewage treatment plants. The quality of effluent released to natural waters requires scrutiny, since some effluent from sewage treatment plants ending up in water bodies has been shown to affect fish and other organisms living in natural waters through pharmaceuticals (Brodin et al. 2013), microplastics (e.g. Magnusson and Norén 2014) and antibiotic-resistant bacteria (Berglund et al. 2015) In natural waters, degradation of organic pollutants is much slower than in soil, where many kinds of microorganisms degrade water-soluble organic compounds relatively quickly (Neumann et al. 2014).

Finally, more than one-third of the human population in developing countries lacks access to basic sanitation, and as much as 90 % of all wastewater worldwide may be discharged untreated into rivers, lakes or oceans, affecting human health and ecosystems (Corcoran et al. 2010). In these countries, major investment in sewage treatment is urgently needed to reach the sustainable development goal of access to water and sanitation for all set by the United Nations.
